# Association between cognitive functioning and health-related quality of life and its mediation by depressive symptoms in older patients with kidney failure

**DOI:** 10.1007/s40620-024-02095-3

**Published:** 2024-09-26

**Authors:** Imre Demirhan, Mathijs van Oevelen, Zeinab Skalli, Carlijn G. N. Voorend, Simon P. Mooijaart, Yvette Meuleman, Marianne C. Verhaar, Willem Jan W. Bos, Marjolijn van Buren, Alferso C. Abrahams, DIALOGICA study group, P Leurs, J B van der Net, T T Cnossen, K Goossens, A Neradova, F van Breda, M Eshuis, K L W Bunthof, R ter Meulen, R A G J Dam, C J A M Konings, A van Eck van der Sluijs, S J J Logtenberg, D Severs, H A Polinder-Bos, A H Boonstra, J van der Leeuw, Y M Vermeeren, N H Hommes, M van Buren, M A Siezenga, M M S Golüke, M H Kallenberg, E K Hoogeveen, A P M Kerckhoffs, T Cornelis, S Boorsma, H Bouwsma, W M Michels, R M A van den Dorpel, B Hoekstra, J M H Joosten, E J R Litjens, A B Kramer, A Kuijper, R J Bosma, M D M Romijn, A Y Adema, A Bontemps-Visser, B van Dam, W van der Meijden, H Boom, G van Kempen, H H T I Klein, W J W Bos, J D Snoep, M H P J Schuurmans, F L Nauta, C F M Franssen, A Diepenbroek, A C Abrahams, F M Molenaar, K François, I Wauters, M Krekels, F Plum

**Affiliations:** 1 Department of Nephrology and Hypertension, University Medical Center Utrecht, Utrecht, The Netherlands; 2 Department of Internal Medicine (Nephrology), Leiden University Medical Center, Leiden, The Netherlands; 3 Department of Internal Medicine, Section of Gerontology and Geriatrics, Leiden University Medical Center, Leiden, The Netherlands; 4 Department of Clinical Epidemiology, Leiden University Medical Center, Leiden, The Netherlands; 5 Department of Internal Medicine, St. Antonius Hospital, Nieuwegein, The Netherlands; 6 Department of Internal Medicine, Haga Hospital, The Hague, The Netherlands

**Keywords:** Quality of life, Cognitive functioning, Older patients, Kidney failure

## Abstract

**Background:**

Impaired cognition, poor health-related quality of life (HRQoL) and depressive symptoms are common in older patients with kidney failure. Understanding what influences HRQoL is important, as older patients regard HRQoL as a health priority. This study examines whether cognitive functioning is associated with HRQoL and whether depressive symptoms mediate this effect in older patients with kidney failure.

**Methods:**

Outpatients aged ≥ 65 years from 35 Dutch and Belgian hospitals with eGFR 20–10 mL/min/1.73 m^2^ were included from the ongoing DIALOGICA study. Cognitive functioning was assessed using the Montreal Cognitive Assessment. Depressive symptoms were screened with 2 Whooley Questions and thereafter assessed with the 15-item Geriatric Depression Scale. HRQoL was assessed using the 12-item Short-Form Health Survey. To assess whether cognitive functioning is associated with HRQoL, cross-sectional multivariable linear regression analyses were performed. Subsequent mediation analyses were performed with PROCESS using the product method.

**Results:**

In total, 403 patients were included, with a mean age of 76.5 years (SD 5.8) and estimated glomerular filtration rate (eGFR) of 14.5 mL/min/1.73 m^2^ (SD 3.0). Cognitive functioning was associated with mental HRQoL (adjusted *β* 0.30, 95% CI 0.05;0.55) but not physical HRQoL (adjusted *β* 0.18, 95% CI -0.09;0.44). This effect is mediated by depressive symptoms (adjusted *β* 0.14, 95% CI 0.04;0.25).

**Conclusion:**

Lower cognitive functioning was negatively associated with mental HRQoL, which was mediated by depressive symptoms in older patients with kidney failure. Future research should explore whether cognitive interventions and treatment of depression improve HRQoL in this vulnerable patient population.

**Graphical Abstract:**

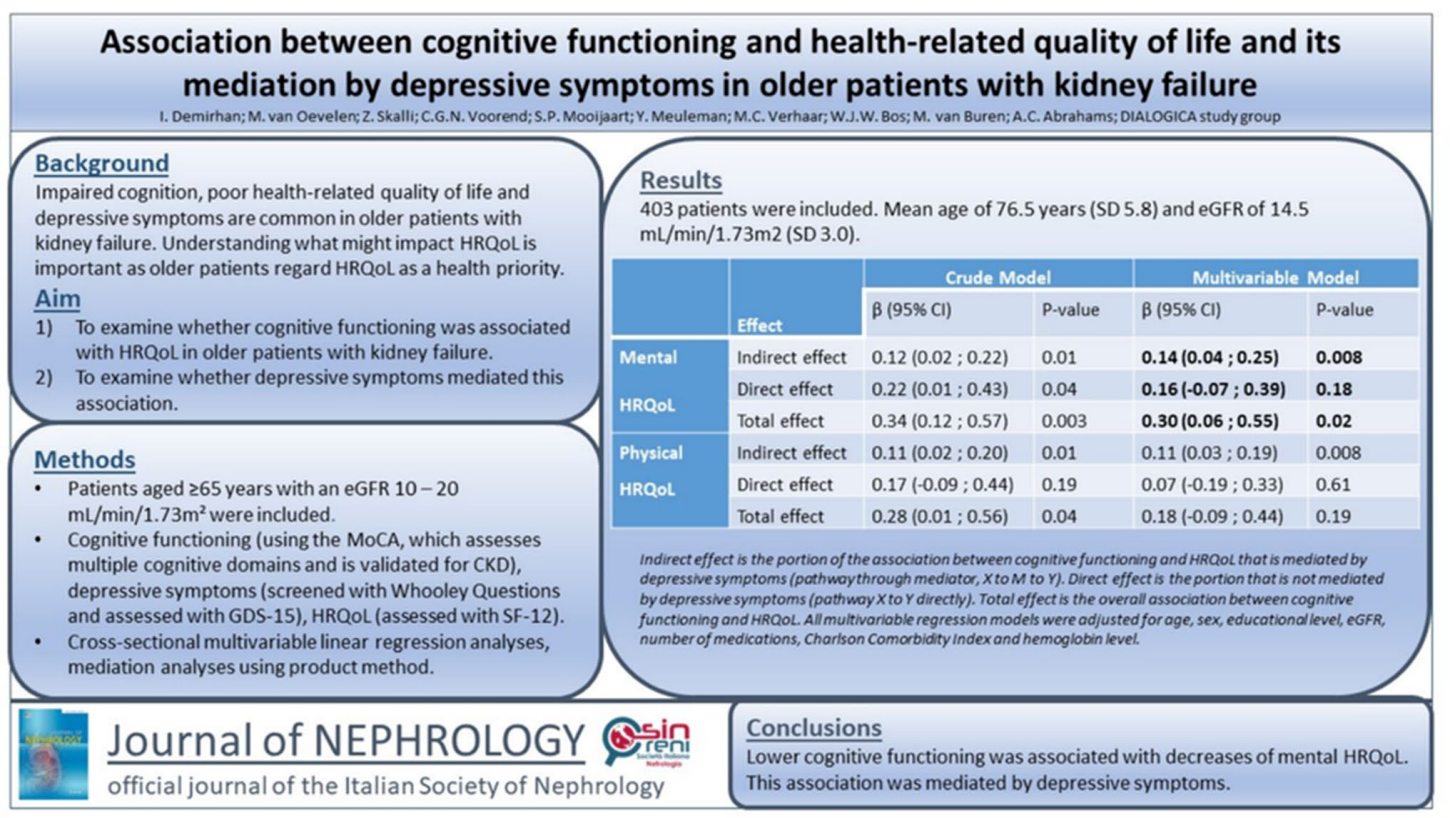

**Supplementary Information:**

The online version contains supplementary material available at 10.1007/s40620-024-02095-3.

## Introduction

Older patients with chronic kidney disease (CKD) often experience poor health-related quality of life (HRQoL) [1]. Maintaining independence and HRQoL is prioritized over survival by most older patients with CKD [2]. In addition, poor HRQoL is associated with adverse clinical outcomes, including mortality and hospitalization [3]. In recent years, HRQoL has become more important when setting treatment goals, and there is growing emphasis on the incorporation of patient preferences in medical decision making in CKD [4]. Thus, improving our understanding of what might impact HRQoL in older patients with kidney failure is vital.

In addition to poor HRQoL, older patients with CKD often experience cognitive impairment as well, with a high prevalence of 13–70% in patients with CKD [5]. Cognitive impairment influences (the patient’s ability to participate in) medical decisions and associates with poor clinical outcomes such as hospitalization and mortality [6]. Despite the high prevalence of cognitive impairment, it is unclear whether cognitive functioning is associated with HRQoL in older patients with kidney failure. Previous studies examining cognitive functioning and HRQoL in CKD did not specifically examine older patients with kidney failure, included a wide range of CKD stages including dialysis, and showed conflicting results [7–10]. Moreover, previous studies in several non-CKD populations showed that depressive symptoms mediate the effect of cognitive functioning on HRQoL [11–14]. To date, it remains unclear whether depressive symptoms mediate the association between cognitive functioning and HRQoL in older patients with CKD.

Therefore, the aim of this study is to examine the association between cognitive functioning and HRQoL, specifically in older patients with kidney failure, and to explore the potential mediating role of depressive symptoms therein.

## Methods

### Study design and participants

The DIALysis or not: Outcomes in older kidney patients with GerIatriC Assessment (DIALOGICA) study is an ongoing multicenter prospective observational study in 40 hospitals in the Netherlands and Belgium. DIALOGICA compares HRQoL, clinical outcomes and healthcare costs of patients choosing dialysis versus conservative care. Patients are eligible for inclusion if aged ≥ 65 years, with an estimated glomerular filtration rate (eGFR) between 20 and 10 mL/min/1.73 m^2^, determined by the Chronic Kidney Disease Epidemiology Collaboration (CKD-EPI) equation, or an average urea and creatinine clearance in 24-h urine between 20 and 10 mL/min. Prior to study participation, patients provided written informed consent. Data are collected at inclusion and subsequently yearly, and after reaching eGFR ≤ 10 mL/min/1.73 m^2^ or starting kidney replacement therapy (KRT). This includes a nephrology-tailored geriatric assessment (i.e.; a consensus-based set of instruments and questionnaires assessing somatic, mental, physical and social geriatric domains) [15]. Clinical and demographic data are obtained from electronic patient records and questionnaires. A complete description of the methodology of DIALOGICA can be found in the previously published rationale paper [16]. The study is conducted according to the principles of the Declaration of Helsinki and the ICH-GCP guidelines and approved by the Medical Ethics Review Committee South-West Holland (METC Zuidwest Holland, reference number 19–071). The first patient was included on May 13th, 2020. For the present cross-sectional study, we used baseline data of patients who were included prior to September 23rd, 2023, and who fully completed a cognitive assessment and questionnaires assessing HRQoL and depressive symptoms. Patient characteristics at baseline were derived from questionnaires and electronic patient records. Results are reported in accordance with the STrengthening the Reporting of OBservational studies in Epidemiology (STROBE) guidelines, as per the fillable STROBE Checklist for cross-sectional studies.

### Measurements

#### Cognitive functioning

Cognitive functioning was assessed using the Montreal Cognitive Assessment, with a fully completed Montreal Cognitive Assessment score ranging from 0 to 30. A lower score indicates worse cognitive functioning, and a score of ≤ 25 indicates cognitive impairment. The Montreal Cognitive Assessment consists of 12 separate tasks assessing different cognitive domains, including memory, executive functioning, attention, language, visuospatial functioning and orientation [17]. The Montreal Cognitive Assessment is frequently used for patients with CKD and is considered superior to the Mini-Mental State Examination as it includes assessment of executive functioning [18].

#### Health-related quality of life

HRQoL was assessed using the 12-Item Short-Form Health Survey version 2, which has been validated to measure HRQoL in CKD populations [19]. The 12-item Short-Form Health Survey yields two scores, namely the Mental Component Summary score and the Physical Component Summary score. The mental component summary score comprises vitality, social functioning, role limitations due to emotional problems, and mental health—all reflecting mental HRQoL. The physical component summary score comprises physical functioning, role limitations due to physical problems, bodily pain, and general health—all reflecting physical HRQoL [20]. The mental component summary and physical component summary scores can range from 0 to 100, with higher scores representing a better HRQoL.

#### Depressive symptoms

The presence of depressive symptoms was assessed in two steps using two separate questionnaires. First, patients answered the Whooley Questions, a two-item screening instrument which inquires about depressed mood and anhedonia [21]. Second, only in case of at least one positive answer on the Whooley Questions was the 15-item Geriatric Depression Scale assessed, resulting in a score ranging from 0 to 15 with higher scores indicating more depressive symptoms [22]. If both Whooley Questions were answered negatively, the 15-item Geriatric Depression Scale score was assumed to be zero. The 15-item Geriatric Depression Scale has shown to perform well as a screening tool for depression in older patients with kidney failure [23].

### Statistical analyses

Frequencies and percentages for categorical data and means with standard deviations (SD) or medians with interquartile range (IQR) for continuous data of the patient characteristics were calculated and shown, depending on data distribution. For interpretation purposes, the prevalence of cognitive impairment was calculated using the cut-off of the Montreal Cognitive Assessment (score ≤ 25).

To assess whether cognitive functioning was associated with HRQoL, multivariable linear regression analyses were performed using the Montreal Cognitive Assessment as determinant and the mental component summary and physical component summary as outcomes. Analyses were adjusted for potential confounders and added in several models. Model 1 comprised cognitive functioning (crude) alone, model 2 added age, sex and educational level, and model 3 additionally included eGFR, number of medications, Charlson Comorbidity Index and hemoglobin level.

Second, the mediating role of depressive symptoms on the association between cognitive functioning and HRQoL was explored. For this purpose, the PROCESS macro version 4.2 by Hayes in R was used, applying the product method to assess the direct and indirect effects on HRQoL while adjusting for the aforementioned confounders [24]. For all mediation models, 5000 bootstrapped samples were used.

Regression coefficients (*β*), *p*-values and 95% confidence intervals (CIs) were calculated for all analyses. A *p*-value lower than 0.05 was considered statistically significant. Missing values were handled using multiple imputation in SPSS using ten iterations, with none of the variables missing over 2% of the values. Pooling of the mediation results was performed using the MICEADDS package in R by applying Rubin’s Rules. A figure was created to visualize the hypothesized (in)direct pathways between cognitive functioning, HRQoL and depressive symptoms. Data analyses were performed using IBM SPSS Statistics version 29 and R version 4.3.2.

#### Sensitivity analyses

Baseline characteristics of excluded patients were compared with those of included patients to explore potential selection bias. To this end, significance of differences were tested using the *T*-test, Chi-Square test and Mann–Whitney U test when appropriate. To assess the potential effect of multiple imputation, a complete case analysis was performed using the non-imputed dataset.

## Results

On September 23rd, 2023, 595 patients were included in DIALOGICA. On that date, 403 patients had fully completed the Montreal Cognitive Assessment, the 12-item Short-Form Health Survey and the Whooley Questions/15-item Geriatric Depression Scale and were thus included in this study. Patient characteristics are shown in Table 1. Most patients were male (71.5%), had a mean age of 76.5 (SD 5.8) years and a mean eGFR of 14.5 (SD 3.0) mL/min/1.73 m^2^. In total, 222 patients (55.1%) had cognitive functioning scores on the Montreal Cognitive Assessment indicative of cognitive impairment. Overall, patients had a better mental HRQoL than physical HRQoL, with a mean mental component summary score of 52.8 (SD 9.2) and physical component summary score of 39.2 (SD 11.0).

**Table 1:** Patient characteristics

Characteristics	Total cohort (*N* = 403)
Age in years, mean (SD)	76.5 (5.8)
Male sex, n (%)	288 (71.5)
eGFR in mL/min/1.73 m^2^, mean (SD)	14.5 (3.0)
Primary kidney disease, *n* (%)	
Diabetic kidney disease	82 (20.3)
Glomerulonephritis	23 (5.7)
Hypertensive nephropathy	80 (19.9)
Polycystic kidney disease	15 (3.7)
Pyelonephritis	3 (0.7)
Renal vascular disease	76 (18.9)
Other	105 (26.1)
Unknown	19 (4.7)
Number of different medications, mean (SD)	10.9 (4.3)
Hemoglobin level in g/dL, mean (SD)	11.8 (1.5)
Presence of anemia, n (%)^a^	295 (73.2)
Marital status, *n* (%)	
Unmarried	39 (9.7)
Married and/or living together	260 (64.5)
Divorced	28 (6.9)
Widow(er)	76 (18.9)
Living situation, *n* (%)	
Independent	151 (37.5)
With partner	232 (57.6)
With other family members	14 (3.5)
Nursing home	6 (1.5)
Educational level^a^, *n* (%)	
Lower	142 (35.2)
Intermediate	138 (34.2)
Higher	123 (30.5)
Charlson Comorbidity Index, median (IQR)	4 (3—5)
Clinical frailty scale, median (IQR)	3 (2—4)
Malnourished or at risk of malnourishment, *n* (%)^c^	105 (26.1)
Smoking status, *n* (%)	
Current smoker	32 (7.9)
Previous smoker	257 (63.8)
Alcohol use status, *n* (%)	
Drinks currently	181 (44.9)
Prior drinker	60 (14.9)
MoCA score, median (IQR)	25 (22–27)
MoCA score ≤ 25, *n* (%)	222 (55.1)
MCS score, mean (SD)	52.8 (9.2)
PCS score, mean (SD)	39.2 (11.0)
Whooley Questions with one or two ‘yes’ answers, *n* (%)	117 (29.0)
GDS-15 score, median (IQR)	0 (0–1)
GDS-15 score ≥ 5, *n* (%)	40 (9.9)

Missing values that were imputed included: Primary kidney disease (1.5%), number of medications (1.7%), Hemoglobin (2.0%), marital status (1.0%), living situation (1.0%), educational level (1.7%), nutritional status (2.0%), smoking status (1.0%), and alcohol use (2.0%)

*eGFR* estimated glomerular filtration rate, *MoCA* montreal cognitive assessment, *MCS* mental component summary, *PCS* physical component summary, *GDS* geriatric depression scale. None of these questionnaires had missing values. MoCA score of ≤ 25 implies cognitive impairment, GDS-15 score of ≥ 5 indicates a potential depression

^a^Anemia defined as hemoglobin of < 13 g/dL in males, < 12 g/dL in females according to the KDIGO guidelines. ^b^Based on the Verhage education classification. ^c^Based on the Mini Nutritional Assessment Short-Form

### Cognitive functioning and health-related quality of life

The results of the crude and multivariable linear regression analyses regarding the association between cognitive functioning and HRQOL are presented in Table 2. The crude analyses showed that cognitive functioning was associated with both mental HRQoL (*β* 0.35, 95% CI 0.12;0.57) and physical HRQoL (*β* 0.29, 95% CI 0.01;0.56). After adjusting for potential confounders, the effect of cognitive functioning remained statistically significant with a similar effect size for mental HRQoL (adjusted *β* 0.30, 95% CI 0.05;0.55). Thus, every 1-point decrease in cognitive functioning results in a 0.30 decrease in mental HRQOL. However, the influence on physical HRQoL became weaker and lost significance (adjusted *β* 0.18, 95% CI − 0.09;0.44).

**Table 2:** Association between cognitive functioning and mental and physical HRQoL

	Model	Mental HRQoL^b^				Physical HRQoL^b^			
		*β*	95% CI	*P*-value	*R*^2^	*β*	95% CI	*P*-value	*R*^2^
Cognitive functioning^a^	1	0.35	0.12; 0.57	0.003	0.02	0.29	0.01; 0.56	0.04	0.01
	2	0.31	0.06; 0.55	0.01	0.04	0.25	-0.04; 0.54	0.10	0.06
	3	0.30	0.05; 0.55	0.02	0.05	0.18	-0.09; 0.44	0.19	0.22

Model 1: Cognitive functioning

Model 2: Model 1, additionally adjusted for age, sex, and educational level

Model 3: Model 2, additionally adjusted for eGFR, number of medications, Charlson Comorbidity Index and hemoglobin level

^a^Cognitive functioning measured using the MoCA. Scores range from 0 to 30

^b^Assessed with the SF-12 (i.e. the MCS and PCS scores. Scores range from 0 to 100)

### Mediating role of depressive symptoms

**Fig. 1:**
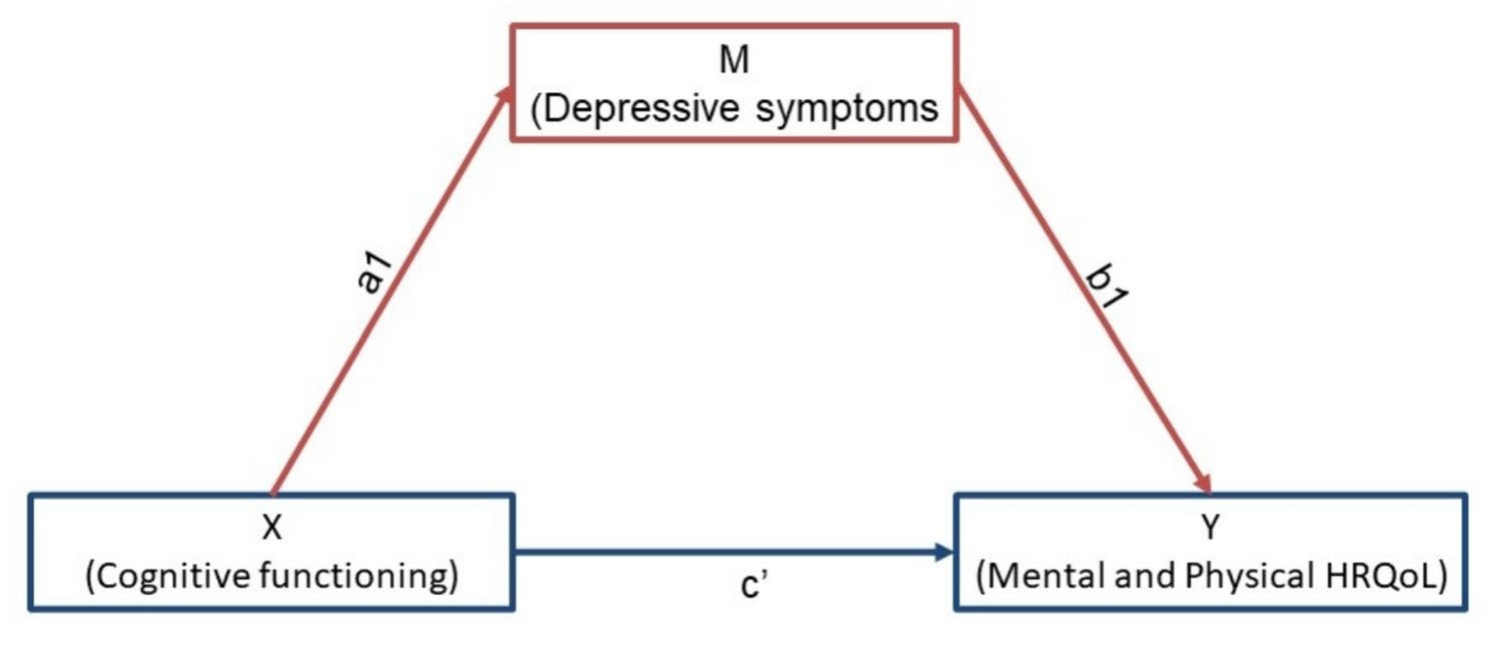
Hypothesized model of the association between cognitive functioning and mental & physical HRQoL mediated by depressive symptoms. Pathways: indirect effect is pathway X to M (mediator) to Y (a1 + b1). Direct effect is pathway X to Y directly (c’). Total effect is both pathways combined (a1 + b1 + c’)

In Fig. 1, the hypothesized (in) direct pathways between cognitive functioning and mental and physical HRQoL are visualized with depressive symptoms as mediator. In Table 3, the results of the crude and multivariable mediation analyses are presented. The results showed that, after adjusting for potential confounders, the association between cognitive functioning and mental HRQoL was mediated by depressive symptoms (adjusted *β* 0.14, 95% CI 0.04;0.25). This mediation effect implies that reductions in cognitive functioning were associated with increases in depressive symptoms, and consequently reductions in mental HRQoL.

**Table 3:** Mediating role of depressive symptoms on the association between cognitive functioning and mental & physical HRQoL

	Effect	Crude model		Multivariable model	
		*β* (95% CI)	*P* value	*β* (95% CI)	*P* value
Mental HRQoL	Indirect effect	0.12 (0.02; 0.22)	0.01	0.14 (0.04; 0.25)	0.008
	Direct effect	0.22 (0.01; 0.43)	0.04	0.16 (− 0.07; 0.39)	0.18
	Total effect	0.34 (0.12; 0.57)	0.003	0.30 (0.06; 0.55)	0.02
Physical HRQoL	Indirect effect	0.11 (0.02; 0.20)	0.01	0.11 (0.03; 0.19)	0.008
	Direct effect	0.17 (− 0.09; 0.44)	0.19	0.07 (− 0.19; 0.33)	0.61
	Total effect	0.28 (0.01; 0.56)	0.04	0.18 (− 0.09; 0.44)	0.19

All multivariable regression models were adjusted for age, sex, educational level, eGFR, number of medications, Charlson Comorbidity Index and hemoglobin level. Indirect effect is pathway a1 + b1, direct effect is pathway c’, and total effect is a1 + b1 + c’ (also see Fig. 1)

### Sensitivity analyses

Differences in baseline characteristics between included and excluded patients are shown in supplemental Table 1. Included patients were younger (*p* = 0.01), had a lower educational level (*p* = 0.03) and were less frail according to the Clinical Frailty Scale (*p* = 0.005).

Supplementary Table 2 shows the results of both crude and multivariable regression analyses for cognitive functioning and HRQoL using the original unimputed dataset, which yielded similar results.

Supplementary Table 3 shows the results of the mediation analyses using the original unimputed dataset for complete case analysis, again showing consistent results.

## Discussion

This study showed that lower cognitive functioning was negatively associated with the mental HRQoL of older patients with kidney failure. This effect was significantly mediated by depressive symptoms, meaning reductions in cognitive functioning were associated with increases in depressive symptoms with subsequent reductions in mental HRQoL. No significant association between cognitive functioning and physical HRQoL was found. The prevalence of cognitive impairment in our cohort was high (55.1%).

Only a few recent studies have investigated cognitive functioning and mental HRQoL in CKD [7, 8, 10]. In these studies, the populations were more heterogeneous in age (also including younger patients) and kidney function compared to our cohort, and mediation by depressive symptoms was not explored. Similar to our findings, Thancharoen et al. found an association between cognitive impairment and anxiety/depression scores on the EQ-5D-5L in a cohort of 379 patients [10]. It should be noted that this study included younger patients (mean 65.7 years) with a higher eGFR (mean 22.8 mL/min/1.73 m^2^) compared to our cohort. Furthermore, the Mini-Mental State Examination was used instead of the Montreal Cognitive Assessment, which does not measure executive functioning. This perhaps contributes to the lower cognitive impairment prevalence of 15.8% [10, 18]. In two smaller studies of Seidel et al. and Greinert et al., no significant association was found between cognitive functioning and mental HRQoL [7, 8]. This was probably due to the smaller and younger study populations (119 patients with mean age of 61.5 years, 62 patients with median age 66 years, respectively), wider ranges of eGFR (mean eGFR of 28.2 mL/min/1.73 m^2^, median eGFR of 28.4 mL/min/1.73 m^2^, respectively) and due to substantially different cognitive tests used.

The significant association between cognitive functioning and mental HRQoL we found in our cohort may be due to several factors. Older patients with CKD usually experience better mental HRQoL compared to younger patients, potentially partly due to better psychological/mental resilience and emotional regulation [25]. It is possible that cognitive dysfunction in this group can negatively influence both psychological resilience and emotional well-being, which in turn can lead to depressive symptoms [26, 27]. Our finding that depressive symptoms mediate the effect of cognitive functioning on mental HRQoL is therefore logical and in line with findings from previous studies in non-CKD populations, e.g. with Parkinson’s disease [11], older patients with schizophrenia [12], multiple sclerosis [13] and breast cancer [14].

The association between cognitive functioning and physical HRQoL lost significance and effect size after adjusting for the potential confounders age, sex, educational level, eGFR, number of medications, Charlson Comorbidity Index and hemoglobin level. It therefore seems that factors other than cognitive functioning, perhaps physical determinants, potentially affect physical HRQoL in older patients with kidney failure. As with mental HRQoL, only a few studies have previously examined cognitive functioning and physical HRQoL, however not specifically in older patients with kidney failure. Similar to our findings, Seidel et al. found no significant association between cognitive functioning and physical HRQoL after adjusting for confounders [7]. Greinert et al. found significant differences in physical HRQoL between groups with different cognitive performance, but did not adjust for confounders [8]. Harhay et al. found no lower physical HRQoL in patients with cognitive impairment assessed with the Mini-Mental State Examination, examining a cohort of 630 younger patients (median age 60 years) and finding a lower cognitive impairment prevalence of 18.6% [9].

Diagnosing cognitive impairment in CKD populations may be challenging, as not all global cognitive functioning tests include executive functioning. Since patients with CKD and cognitive impairment typically exhibit executive dysfunction, the Montreal Cognitive Assessment may be more suitable than the Mini-Mental State Examination and other tests lacking assessment of executive functioning [18]. For that reason, the Montreal Cognitive Assessment was specifically chosen over the Mini-Mental State Examination when the nephrology-tailored geriatric assessment was designed and used in DIALOGICA [15, 16]. Our use of the Montreal Cognitive Assessment might also partly explain the differences with the abovementioned other studies where different tests were used, and could explain the high prevalence of cognitive impairment we found (55.1%) [7–10].

The findings of this study contribute to our understanding of what might impact HRQoL in older patients with kidney failure. This may help both patients and nephrologists, as HRQoL is an important health priority for older patients with CKD which guides medical treatment decisions [2, 6]. Screening for cognitive impairment is often not routinely performed but is standard in the nephrology-tailored geriatric assessment [15, 18]. This may help to identify older patients with kidney failure who could benefit both from (cognitive) interventions to preserve cognitive functioning and from further guidance [18, 28]. As cognitive functioning was associated with mental HRQoL with mediation by depressive symptoms, future research should assess whether cognitive interventions and available treatments of depressive symptoms (with medication or psychological interventions) improve HRQoL in older patients with kidney failure. This may be likely, as there is some evidence that HRQoL can be improved by treating depression in kidney failure [29]. It should be noted that only a small effect size was detected: a 1 point decrease in cognitive functioning was associated with a decrease of 0.3 point in mental HRQoL. Thus, a clinically relevant difference of 3 points of mental HRQoL would require a 10 point difference in cognitive functioning [30]. This small effect size could partly be explained by a ceiling effect, as 5.7% of patients scored the maximum score on the Montreal Cognitive Assessment and a high median (IQR) of the Montreal Cognitive Assessment was noted.

The association between cognitive functioning and HRQoL was explored in a large cohort with a high prevalence of cognitive impairment, i.e. older patients with kidney failure. Another strength was the use of the Montreal Cognitive Assessment, which is a quick and suitable assessment tool for older patients with CKD. Moreover, by using mediation analyses, further insight was gained into how cognitive functioning was associated with HRQoL, and we indeed found depressive symptoms to be a mediator. Furthermore, robustness of our findings was evaluated by adjusting for relevant confounders and performing sensitivity analyses. Finally, the generalizability of our findings has been improved by including patients from 35 hospitals in the Netherlands and Belgium.

This study also has some limitations. First, our study had a cross-sectional design, therefore limiting the ability to make causal inferences. Thus, further research would be needed to assess whether changes in HRQoL are caused by (changes in) cognitive functioning and related depressive symptoms. Second, the median for the Montreal Cognitive Assessment scores was high, which may have led to a ceiling effect, and therefore an underestimation of the association with HRQoL. Third, our two-step approach for depressive symptoms (the 15-item Geriatric Depression Scale was only assessed if either or both of the screening Whooley Questions were answered with 'yes') may have resulted in an underestimation of depressive symptoms. Fourth, there were some significant differences in patient characteristics between included and excluded patients, which may imply a risk of selection bias. More specifically, the excluded patients were older, with different educational levels and more frail, which may lead to a further underestimation of our effects as these patients are likely to have lower cognitive functioning.

## Conclusion

In conclusion, cognitive functioning was associated with mental but not physical HRQoL in older patients with kidney failure. This effect was mediated by depressive symptoms. These findings may increase patients’ and healthcare professionals’ understanding of potential factors affecting HRQoL, and highlight the need for awareness of cognitive impairments, depressive symptoms, and further investigation of interventions to improve HRQoL in this vulnerable population.

## Supplementary Information

Below is the link to the electronic supplementary material.

Supplementary file1 (DOCX 24 KB)

## Data Availability

The data that support the findings of this study are available from the corresponding author upon reasonable request.
