# Supplementary material for: Association between cognitive functioning and health-related quality of life and its mediation by depressive symptoms in older patients with kidney failure
Source: J Nephrol. 2024 Sep 26;37(7):1939–48. doi: 10.1007/s40620-024-02095-3 (PMC11519205; doi:10.1007/s40620-024-02095-3)
Supplement: Supplementary file 1 — Supplementary file1 (DOCX 24 KB) [file 40620_2024_2095_MOESM1_ESM.docx]

**Supplemental Table 1: Patient characteristics of included and excluded patients**

| Characteristics | Included patients (N=403) | Excluded patients (N=192) | P-value^a^ |
| --- | --- | --- | --- |
| Age in years, mean (SD) | 76.5 (5.8) | 77.8 (5.7) | 0.01 |
| Male sex, n (%) | 288 (71.5) | 126 (65.6) | 0.15 |
| eGFR in mL/min/1.73m², mean (SD) | 14.5 (3.0) | 14.5 (3.6) | 0.96 |
| Primary kidney disease, n (%) |  |  | 0.28 |
| Diabetic kidney disease | 82 (20.3) | 41 (21.4) |  |
| Glomerulonephritis | 23 (5.7) | 9 (4.7) |  |
| Hypertensive nephropathy | 80 (19.9) | 39 (20.3) |  |
| Polycystic kidney disease | 15 (3.7) | 12 (6.3) |  |
| Pyelonephritis | 3 (0.7) | 4 (2.1) |  |
| Renal vascular disease | 76 (18.9) | 27 (14.1) |  |
| Other | 105 (26.1) | 43 (22.4) |  |
| Unknown | 19 (4.7) | 17 (8.9) |  |
| Number of medications, mean (SD) | 10.9 (4.3) | 10.9 (4.3) | 0.92 |
| Hemoglobin level in g/dL, mean (SD) | 11.8 (1.5) | 11.8 (1.6) | 0.67 |
| Presence of anemia, n (%)^b^ | 295 (73.2) | 142 (74.0) | 0.81 |
| Marital status, n (%) |  |  | 0.72 |
| Unmarried | 39 (9.6) | 25 (13.0) |  |
| Married and/or living together | 260 (64.7) | 101 (52.6) |  |
| Divorced | 28 (6.9) | 21 (10.9) |  |
| Widow(er) | 76 (18.8) | 45 (23.4) |  |
| Living situation, n (%) |  |  | 0.16 |
| Independent | 151 (37.5) | 70 (36.5) |  |
| With partner | 232 (57.6) | 89 (46.4) |  |
| With other family members | 14 (3.5) | 8 (4.2) |  |
| Nursing home | 6 (1.5) | 25 (13.0) |  |
| Educational level, n (%)^c^ |  |  | 0.03 |
| Lower | 142 (35.2) | 81 (42.2) |  |
| Intermediate | 138 (34.3) | 58 (30.2) |  |
| Higher | 123 (30.5) | 53 (27.6) |  |
| Charlson Comorbidity Index, median (IQR) | 4 (3-5) | 4 (3-5) | 0.90 |
| Clinical frailty scale, median (IQR) | 3 (2 - 4) | 3.75 (3 - 5) | 0.005 |
| Malnourished or at risk of malnourishment, n (%)^d^ | 105 (26.1) | 53 (27.6) | 0.91 |
| Smoking status, n (%) |  |  | 0.78 |
| Current smoker | 32 (7.9) | 23 (12.0) |  |
| Previous smoker | 257 (63.8) | 108 (56.3) |  |
| Alcohol use status, n (%) |  |  | 0.30 |
| Drinks currently | 181 (44.9) | 73 (38.0) |  |
| Prior drinker | 60 (14.9) | 32 (16.7) |  |

eGFR: estimated glomerular filtration rate. ^a:^ Significance was assessed using the T-test for differences in means, Chi-Square test for categorical variables and the Mann-Whitney U test for differences in medians. ^b^Anemia defined as hemoglobin of <13 g/dL in males, <12 g/dL in females according to the KDIGO guidelines.^c:^ Based on the Verhage education classification. ^d^Based on the Mini Nutritional Assessment Short-Form

**Supplemental Table 2: Association between cognitive functioning and mental & physical HRQoL (N=387)**

|  |  | Mental HRQoL^b^ | | | | Physical HRQoL^b^ | | |  |
| --- | --- | --- | --- | --- | --- | --- | --- | --- | --- |
|  | Model | β | 95% CI | p | R^2^ | β | 95% CI | p | R^2^ |
| Cognitive functioning^a^ | 1 | 0.35 | 0.12 ; 0.57 | 0.003 | 0.022 | 0.29 | 0.01 ; 0.56 | 0.04 | 0.01 |
|  | 2 | 0.31 | 0.06 ; 0.56 | 0.014 | 0.037 | 0.23 | -0.06 ; 0.53 | 0.12 | 0.06 |
|  | 3 | 0.31 | 0.06 ; 0.57 | 0.02 | 0.05 | 0.16 | -0.11 ; 0.43 | 0.25 | 0.22 |

Model 1: Cognitive functioning

Model 2: Model 1, additionally adjusted for age, sex and educational level

Model 3: Model 2, additionally adjusted for eGFR, number of medications, Charlson Comorbidity Index and hemoglobin level

^a^: Cognitive functioning is measured using the MoCA. Scores range from 0 to 30.

^b^: Assessed with the SF-12 (i.e. the MCS and PCS. Scores range from 0 to 100)

**Supplemental table 3: Mediating role of depressive symptoms on the association between cognitive functioning and mental & physical HRQoL (N=387)**

|  | Effect | Crude Model | | Multivariable Model | |
| --- | --- | --- | --- | --- | --- |
|  |  | β **(95% CI)** | **P-value** | β **(95% CI)** | **P-value** |
| Mental HRQoL | Indirect effect | 0.12 (0.02 ; 0.22) | 0.01 | 0.15 (0.04 ; 0.26) | 0.007 |
|  | Direct effect | 0.22 (0.01 ; 0.43) | 0.04 | 0.16 (-0.07 ; 0.40) | 0.17 |
|  | Total effect | 0.34 (0.12 ; 0.57) | 0.003 | 0.31 (0.06 ; 0.57) | 0.01 |
| Physical HRQoL | Indirect effect | 0.11 (0.02 ; 0.20) | 0.01 | 0.12 (0.04 ; 0.20) | 0.006 |
|  | Direct effect | 0.17 (-0.09 ; 0.44) | 0.19 | 0.04 (-0.22 ; 0.30) | 0.76 |
|  | Total effect | 0.28 (0.01 ; 0.56) | 0.04 | 0.16 (-0.11 ; 0.43) | 0.25 |

All multivariable regression models were adjusted for sex, age, educational level, eGFR, number of medications, Charlson Comorbidity Index and hemoglobin level. Indirect effect is pathway a1 + b1, direct effect is pathway c’, and total effect is a1+b1+c’ (also see Figure 1).
